# “Your Life, Your Health: Tips and Information for Health and Well-Being”: Development of a World Health Organization Digital Resource to Support Universal Access to Trustworthy Health Information

**DOI:** 10.2196/57881

**Published:** 2025-03-06

**Authors:** Danielle M Muscat, Rachael Hinton, Shyama Kuruvilla, Don Nutbeam

**Affiliations:** 1 Sydney Health Literacy Lab, Sydney School of Public Health, Faculty of Medicine and Health, University of Sydney, Sydney, Australia; 2 World Health Organization, Avenue Appia 20, Geneva, 1202, Switzerland; 3 Sydney School of Public Health, Faculty of Medicine and Health, University of Sydney, Sydney, Australia

**Keywords:** health communication, health literacy, consumer health information, digital health, universal health care

## Abstract

**Background:**

Access to trustworthy, understandable, and actionable health information is a key determinant of health and is an essential component of universal health coverage and primary health care. The World Health Organization has developed a new digital resource for the general public to improve health and well-being across different life phases and to support people in caring for themselves, their families, and their communities. The goal was to make trustworthy health information accessible, understandable, and actionable for the general public in a digital format and at the global scale.

**Objective:**

The aim of this paper was to describe the multistage approach and methodology used to develop the resource *Your life, your health: Tips and information for health and well-being* (hereafter, *Your life, your health*).

**Methods:**

A 5-step process was used to develop *Your life, your health,* including (1) reviewing and synthesizing existing World Health Organization technical guidance, member state health and health literacy plans, and international human rights frameworks to identify priority messages; (2) developing messages and graphics that are accessible, understandable, and actionable for the public using health literacy principles; (3) engaging with experts and stakeholders to refine messages and message delivery; (4) presenting priority content in an accessible digital format; and (5) adapting the resource based on feedback and new evidences.

**Results:**

The *Your life, your health* online resource adopts a life-course approach to organize health information based on priority actions and rights that support peoples’ health and well-being across different life stages and specific health topics. The resource promotes health literacy by offering advice on asking questions to health workers, making informed decisions about personal and family health, and effectively using digital media to obtain reliable health information. Additionally, it reflects the ambitions of the Sustainable Development Goals by providing essential information on the social determinants of health and clarifies the distinct roles of individuals, frontline workers, governments, and the media in promoting and protecting health.

**Conclusions:**

Making health information available—including to the public—is an essential step in strengthening the global health information system. The development process for the *Your life, your health* online resource outlined in this article offers a structured approach to translate technical health guidelines into accessible, understandable, and actionable health information for the general public.

## Introduction

### Background

Access to reliable, understandable, and actionable health information is a determinant of health and an essential component of universal health coverage (UHC) [1-3]. Similar to other social determinants of health, there is significant variability and inequity in access to health information and in peoples’ capacity to retrieve, appraise, and use it in making health-related decisions, also referred to as health literacy [4]. Differences in access to health information and levels of health literacy are observed both within and between countries [5-8].

Digital technology has significantly improved access to health information for many people. Several reviews of digital health interventions have demonstrated the feasibility and potential effectiveness of eHealth and mHealth (mobile health) interventions for a range of health conditions in low-, middle-, and high-income countries [9-13]. However, the digital divide is real and significantly impacts peoples’ access to health information [14] and perpetuates many existing health inequities. Additionally, health information accessed from digital platforms (ie, any electronic communication tool, including websites and social media) is not consistently reliable, understandable, and actionable.

Reliable health information originates from a trusted source and is based on an objective interpretation of available scientific evidence [1]. To be understandable and actionable, members of the public with diverse backgrounds and varying levels of health literacy must be able to understand key messages and identify appropriate actions based on the information presented [15]. However, these fundamental requirements of reliability, understandability, and actionability are not always met. This issue became particularly evident during the COVID-19 pandemic, when unintended misinformation and deliberately circulated disinformation were widely available and often amplified by social media [16-18]. A significant proportion of information sources are also biased, sometimes unconsciously but often deliberately, particularly in commercial communications designed to promote specific products or services [1]. Even when health information originates from a reliable, trustworthy source, it is rarely presented in a manner that most people can understand and act upon when making health-related decisions [19,20].

In response to the global challenge of misinformation and disinformation during the COVID-19 pandemic, the World Health Organization (WHO) developed a Framework for Managing the COVID-19 Infodemic. This framework proposed various actions that WHO member states and the civil society could implement to address the infodemic. This included clear guidance on ensuring that health information was provided in a manner that was “actionable” and “presented in ways that are understood by and accessible to all individuals in all parts of all societies” [21]. This framework recognized the important role of WHO, other United Nations (UN) partners, and civil society organizations in providing reliable health information that is adaptable for use in a wide range of contexts.

Long before this, the 1946 WHO Constitution specified that “informed opinion and active cooperation on the part of the public are of the utmost importance in the improvement of the health of the people” [22]. Despite this historical mandate, WHO has generally limited its health communication activities to providing technical advice and support to member states. These communications are not systematically designed for direct use by the general public [20]. Over the past 25 years, this has been gradually changing and WHO has developed global health information resources that can be accessed and used more directly by the general public, such as WHO’s Facts in Pictures [23], Global Health Days [24], and public health advice on COVID-19 [25]. However, at present, much of WHO’s publicly available information tends to be organized by different health topics or is limited to targeted communication campaigns or events. Therefore, it is not always easily accessible and is not available directly to the general public in a consistent form or integrated manner.

To address this gap, WHO has developed an integrated public health advice resource, *Your life, your health: Tips and information for health and well-being* [2] (hereafter referred to in short form as *Your life, your health*). This new digital resource, specifically developed for the general public, has been designed to make trustworthy health information accessible, understandable, and actionable, contributing toward achieving UHC. The aim of this paper is to describe the multistage process and methodology used to develop this resource.

### Theoretical Framework

The theoretical underpinning for the resource was a UHC and life-course approach to health, based on the WHO Life Course Framework [26]. This framework emphasizes that strategies to achieve UHC must be explicitly people-centered and require integrated, evidence-based approaches across the life course. The WHO’s Thirteen General Programme of Work included a dedicated platform on Improving Human Capital across the Life Course, within which this initiative was situated [27].

Additionally, we applied the principles of Universal Design for Learning (UDL) to the development of the resource. UDL is a theoretical paradigm that informs efforts to promote learning for the greatest possible proportion of a population [28]. It is an inclusive learning environment that is universally designed, considers all relevant dimensions of diversity, and proactively minimizes barriers to learning for all individuals [28]. This perspective recognizes that learning occurs in a dynamic interaction between the individual and learning environment. UDL leverages the flexibility of digital technology to design learning environments that, from the outset, offer options for diverse learner needs, ensuring that educational benefits are more equitably and effectively distributed [28]. In the context of health, health literacy universal precautions similarly assume that all patients and caregivers may have difficulty comprehending health information and recommend communicating in ways that ensure universal understanding [29,30].

## Methods

### Development of the “*Your Life, Your Health”* Resource

A 5-step process was used to develop *Your life, your health* [2]:

- Step 1 – A review and synthesis of existing WHO technical guidance, member state health and health literacy plans, and international human rights frameworks to identify evidence-based health guidance for the public;
- Step 2 – The development of messages and graphics to be accessible, understandable, and actionable for the public through the application of health literacy principles;
- Step 3 – Engagement with experts and other stakeholders to refine messages and message delivery;
- Step 4 – Presentation of priority content in an accessible digital format;
- Step 5 – Adaptation and updates based on feedback and new evidence.

Below, we outline the methods related to the first four steps in detail. Similar to WHO guidelines, the resource will evolve and will be updated as new evidence becomes available. It will also need to be adapted for different country contexts and for different audiences in Step 5.

### Ethical Considerations

Ethical approval was not required as this study is based exclusively on published literature, including global and national strategies and plans, with no data collected from human participants.

### Review of Evidence-Based Interventions and Guidance

#### Overview

The first stage of resource development was focused on identifying reliable health information to be included as the basis for *Your life, your health.* There is an overwhelming volume of health information accessible to most people. We sought to identify evidence–based public health actions that individuals or communities could undertake to care for themselves, their families, and their community—either with or without the support of the health system—to improve health and well-being across different life phases. We then prioritized this information by life phase to reduce the volume of information provided in the resource. While making decisions on priorities, we identified interventions or actions within current global and national health strategies that required or presumed an element of individual, caregiver, family, or community initiative or engagement. Interventions or actions that were exclusively health provider-oriented or related to specialized clinical and surgical interventions and care were mostly excluded from the review process.

To identify priority actions, we purposively searched 21 WHO global health strategies across the life course, and additional strategies, guidance, and evidence compendia suggested by WHO partners. This included strategies for specific target populations and life phases (eg, WHO Global Strategy for Women’s, Children’s and Adolescent’s Health), communicable and noncommunicable diseases (eg, WHO Global Action Plan for the Prevention and Control of Noncommunicable Diseases) and cross-cutting strategies on systems, principles, and determinants (eg, Healthy Systems for Universal Health Coverage: A Joint Vision for Healthy Lives). A full list is available in Table S1 in Multimedia Appendix 1.

We also searched the WHO UHC Compendium of Interventions and the Disease Control Priorities 3rd Edition (DCP3) interventions (community worker and health post levels) [31,32]. The UHC Compendium is a database of health services and intersectoral interventions designed to assist countries in making progress towards UHC. The database includes over 3500 health actions across different health areas. The DCP3 provides an up-to-date comprehensive review of priority health interventions and intersectoral policies to reduce mortality and disability which are evidence-based, scalable, and adaptable in multiple settings. Information from the Compendium and DCP3 was cross-checked against the main global causes of mortality and morbidity at each life stage to ensure that the reviewed evidence-based guidance and interventions addressed these challenges.

Finally, we searched for contemporary national and regional health strategies, action plans, and road maps from all 194 WHO member state countries and 6 regions, with a focus on documents available in English and French. These documents were sourced primarily via WHO’s Country Planning Cycle Database [33]. If a current document was unavailable in the Planning Cycle Database, a search was carried out on Google or PubMed using the following search string: *[((“National”) AND (“Health”)) AND ((“Strategy”) OR (“Road Map”) OR (“Action Plan”)) AND (Name of Country or Region)]*. We considered “contemporary” to mean any strategic document informing planning in a member state beyond 2019. We found relevant information from 84 countries across the 6 WHO regions, which is shown in Multimedia Appendix 2.

The evidence searches, review, and syntheses were led by SK, supported by RH and Global Health Insights, a specialist global health research and consulting collaborative, with experience in evidence synthesis and policy analysis.

#### Synthesis of Evidence

Using the documents identified in the evidence review, we listed the interventions and actions that required or presumed an element of individual, caregiver, family, or community initiative or engagement. Using the content analysis approach described by Mays, Pope, and Popay [34], we deductively classified these actions and interventions according to broad life phases adapted from the WHO Life Course Framework [26]: pregnancy, birth, and after childbirth; newborns and children under 5 years; children aged 5-9 years; adolescents and youth aged 10‐24 years; early and middle adulthood (25‐64 years); and later adulthood (≥65 years).

Within each life phase, interventions and actions were coded into topic areas derived from the broad health topics on the WHO website [35]. As we reviewed the content, we added further health topics until the saturation of categories was achieved. The final categories of health topics are shown in Textbox 1. Content analysis was then applied to identify recurrent themes arising from the tabulated content within each health topic.

Textbox 1. **Final categories of health topics.**

- Care and communication in families
- Nutrition
- Physical activity
- Mental health
- Immunization
- Sexual and reproductive health (including family planning)
- Healthy environments
- Social participation
- Violence and injuries
- Smoking, alcohol, and drugs
- Disabilities
- Connecting to services in health and other sectors
- Emergency illness
- Communicable disease
- Noncommunicable disease

Finally, we applied the international human rights framework and a categorization of human rights by the Sustainable Development Goals to develop a matrix of human rights categorized by life phase (eg, rights related to having children) and cross-cutting rights (eg, the right to health). This approach assured that we moved beyond providing only information that required individual action, to ensure that people are informed about their rights, obligations of the government, and personal responsibilities.

Using this methodology, we had reasonable confidence in the quality and consistency of the evidence and were able to use the findings as the basis for prioritization. To keep the volume of information manageable, we aimed to identify 5 essential health messages for each stage in the life course or specific health topic. A health message refers to actionable information related to health and well-being that individuals or communities can use to care for themselves, their families, and their communities (eg, helping children learn to swim). We identified the messages that had the highest potential for promoting health and those most frequently mentioned across the collated information. Inevitably, some issues of importance to individuals, communities, and countries were not included, and people’s rights to health may vary between countries. We recognize the importance of these contextual considerations and have included them into our pilot testing and adaptation actions as indicated below.

### Application of Health Literacy Principles to Make Content Accessible, Understandable, and Actionable

Following the prioritization of health messages, the next step was to draft the main messages and design the resource’s information architecture in a manner that was optimally accessible, understandable, and actionable. We reviewed the national health and health literacy plans of 84 countries with publicly accessible documentation available in English or French to understand how countries developed public-facing messages that were aligned with WHO’s evidence-based guidance and global strategies. We made selective use of a range of widely used tools and resources to inform decisions about the presentation of information once content was approved within WHO. These included the Health Literacy Universal Precautions Toolkit [36], the CDC Clear Communication Index, Health Literacy Online [37], and the Patient Education Materials Assessment Tool. All content was drafted by a WHO consultant with expertise in health communication, and technical and copy editing, who was trained in health literacy principles.

### Review by Health Specialists and Communication and IT Experts

We developed a web-based prototype of the resource for consultation. Between October 2019 and March 2020, we conducted consultations regarding the draft resource with over 30 experts in health, health literacy, communication, and IT. This included consultation and review across WHO departments, including life course, health workforce, reproductive health and rights, environmental health, health promotion and health literacy, and communications, among others. We sought feedback via an online survey on the content (see Multimedia Appendix 3), particularly on the choice of prioritized actions and rights, and advice on the format and presentation of the messages and potential uses and users. We also sought feedback on various graphics related to the main messages to ascertain the most appropriate format for potential users. Additionally, content experts from WHO departments provided comments on both the simple and more detailed messages relevant to their health topic area to ensure alignment with WHO guidance and existing evidence. Finally, we consulted communication and IT experts within WHO on optimizing the digital platform to support the use and dissemination of the resource, depending on how people use the WHO website. This included the potential for translation of the content into other languages and graphics adaptation for various audiences and communication channels. Feedback was collated and analyzed thematically by the first author and compiled into an action report for WHO to finalize priority content and graphics. In instances where experts had provided conflicting advice, all verbatim feedback provided was included, and final decisions were made by team consensus with all authors.

### Presentation of Priority Content in an Accessible Digital Format

Following the consultation process, the content was revised to incorporate feedback on prioritized actions and rights, while also aiming to maintain a readability grade of 8 or lower (as assessed by the Hemingway Editor). The WHO communications and web team supported the development of the resource for the WHO website.

## Results

### Structure

Through the multistage process outlined above, *Your life, your health*: *Tips and information for health and well-being* online resource was designed to be accessible, understandable, and actionable. This resource has five key sections, accessible via the landing page:

1. Tips and information by life phase
2. How to find and use health information
3. A healthy world
4. Know your rights
5. Other health topics

**Figure 1.**
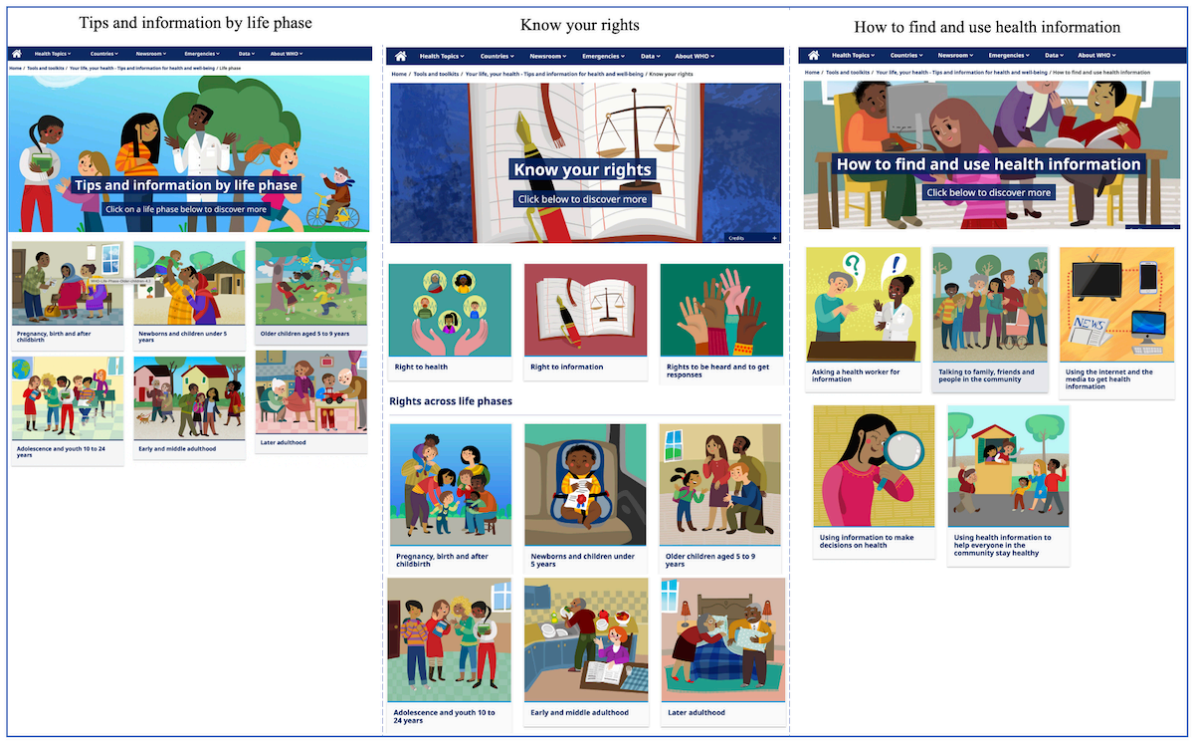
Screenshots representing the structure of three sections of the “Your life, your health: Tips and information for health and well-being” online resource.

This resource adopts WHO’s life-course approach [26] to organize health-related tips and information (Section 1) and rights (Section 4) based on priority actions and rights that support peoples’ health and well-being at different life phases. The consultation process also confirmed that health information should not be focused entirely on individual behaviors and risks, but should reflect how social determinants of health fundamentally influence peoples’ choice of actions and their capacity, more accurately to engage successfully in responding to information. For people in most parts of the world, health decisions and actions are substantially restricted by social and environmental factors beyond their control; peoples’ ability to respond to health information is not universal and equal. In addition, we recognized that people rarely make health decisions in isolation but rather through interaction and support from other people, institutions, organizations, services, and systems. Given this context, Section 3*: A Healthy World* was integrated to reflect the ambitions of the Sustainable Development Goals by providing information on the social determinants of health and clarifying the roles of individuals, frontline workers, governments, and the media in promoting and protecting health. Section 2: *How to find and use health information* was also included to more overtly support users in developing health literacy skills. It includes advice on asking questions to health workers, making informed decisions about personal and family health, and effectively using digital media to access health information (Figure 1).

### Content and Presentation

**Figure 2.**
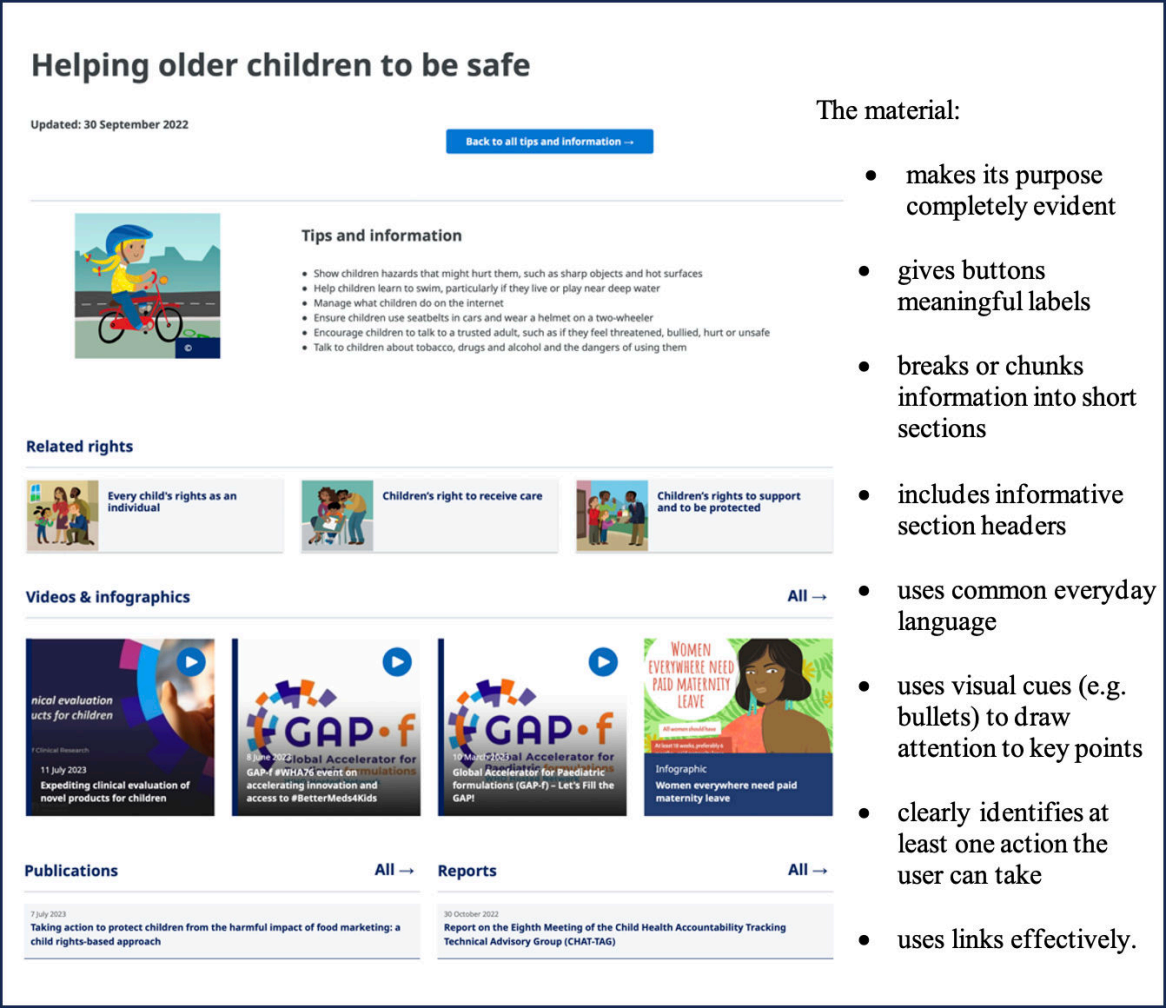
Screenshot from the “Your life, your health: Tips and information for health and well-being” online resource showcasing select design elements from Health Literacy Online and the Patient Education Materials Assessment Tool (PEMAT).

Within each section, we adopted a tiered structure, where information was sequenced from simple to more detailed messages at a level of Grade 8 readability or lower. This would allow the readers to move from the first level consisting of 5 main messages to a second level where each message is broken down into manageable and explicit steps. Further information and more detailed WHO technical content and products could then be made available via links to WHO infographics, videos, and fact sheets depending on needs and preferences. To enhance actionability, each message started with a verb and was worded in a direct way (eg, “Learn about...” “Find out…”). Content cues or markers (eg, bullet points) were also used to highlight or draw attention to each point and 3 styles of frequently used visuals for different life phases and messages (ie, photos, illustrations, and infographics) were developed. Figure 2 shows a screenshot of the *Your life, your health* online resource showcasing select design elements.

### Hosting and Dissemination

As a WHO global good initiative, *Your life, your health* is publicly available to all. The resource is available on the WHO website and is linked to Health Topics [38], for easy access to public health information. To increase accessibility, health advice within the resource is available as downloadable and shareable digital cards with links to expanded messages and resources (See Multimedia Appendix 4). The planned next steps include engaging with the WHO academy to link the resource content to relevant courses and topics for learners from the global health workforce. Other adaptations include integrating *Your life, your health* in home-based records, such as integrated maternal and child health books, which often include health education messages, as well as using content on the WHO Facebook (chatbot) page [39]. Similar to WHO guidelines, the resource will evolve and be updated as new evidence becomes available. Given the synthesis approach adopted in the development of this resource, this will be aligned with updates from the WHO UHC Compendium of Interventions and the Disease Control Priorities. Standard practice steps and a template have been developed to ensure the health literacy principles described here (eg, tiered presentation of information, testing readability) and will continue to provide a guiding framework as new information is integrated. A guidance document has also been developed for WHO departments for developing new content in *Your life, your health* in a standardized format for the WHO website.

## Discussion

As a contribution to improving universal access to trustworthy health information, the WHO has developed an integrated resource of health advice for use by and with the general public. This resource is based on a synthesis of evidence-based guidance derived from the WHO and national-level documents. It is organized to reflect priority actions and rights across different life phases while concurrently seeking to build health literacy skills, and acknowledge and promote awareness of the social determinants of health. As a global resource, *Your life, your health* is intended to complement country-based initiatives such as the US government’s MyHealthFinder or HealthDirect in Australia, which are also evidence-based, easy to read, and action oriented.

Making health information available—including to the public—is an essential step in the global health information system [1]. While developing consumer friendly health information is not a new concept [40,41], there is limited published guidance on how public-facing health information resources are developed, or how to achieve this on a global scale. The development process for *Your life, your health* as outlined in this article, includes reviews of technical guidance and health strategies, the application of health literacy principles, and an expert review and iterative consultation process. It offers a structure that can be adapted for related purposes within and beyond the WHO to translate technical health guidelines into accessible, understandable, and actionable health information for the general public. The development process was built on several well-established health literacy guidelines related to structuring, writing, and visually presenting health information. In addition to the resources referenced above (ie, Health Literacy Universal Precautions Toolkit [32], CDC Clear Communication Index, Health Literacy Online [33]; Patient Education Materials Assessment Tool), there are several other resources more broadly related to universal design. Future research and practice should focus on the selection of resources that are validated and accurate, given inconsistencies across some automated health literacy tools [42]. The selection of guiding resources will also necessarily be context-specific, as mandated guidelines exist across country contexts (eg, US Federal Plain Language Guidelines and Web Content Accessibility Guidelines).

There are strengths and limitations to the development process of WHO’s *Your life, your health* resource. We conducted an evidence synthesis to identify health content, recommendations, and human rights across the life course, including both WHO and external sources. Given the breadth of the resource and scope of health topics included in *Your life, your health,* we reviewed existing global strategies and action plans that are publicly accessible and that already synthesize evidence-based recommendations. By ensuring transparency in the sourcing of evidence and identifying core, consistently advocated health information, we aimed to ensure that the information provided is recognizable, reliable, and trustworthy for those using the resource. This synthesis approach was more efficient than a review of primary studies and allowed for the identification of evidence and recommendations that were consistent across strategies and action plans. While we recognize that this methodology will result in the exclusion of some types of evidence—including newer or emerging evidence which is yet to be incorporated into global strategies—we determined that this approach offered the most secure method for ensuring trust in the information provided, and was supported by expert consultation and review. Additionally, while the exclusion criteria for language were a limitation in our evidence review, the review covered all WHO regions, many of which had national language and English versions available for their strategies.

Our expert review of the content and structure purposefully included diverse expertise, ranging from health literacy to IT; however, it has not yet included community members. Following the publication of this resource, we have initiated a consultative process to iteratively refine its content. This will be followed up by additional co-development and user testing, including with WHO representatives at the regional and country levels, UN agencies, and the general public. Partnering with the general public will result in incremental improvements to the understandability of health information materials [39,43]; the benefits of engaging stakeholders are well-documented and will likely include enhanced relevance, quality, and authenticity, the generation of alternative and innovative ideas and enhanced sustainability of the resource [44]. Adaptation for country-specific contexts will ensure that the resource is culturally acceptable, aligned with local health practices availability of health resources and services, and actionable in a variety of contexts [1]. User testing must include people with variations on literacy, health literacy, and cognitive and functional capacities. The consultations will need to consider feasibility, potential effectiveness, and necessary adaptations of this online digital resource in different settings. It will be important to balance digital engagement with appropriately resourced in-person engagement to ensure that vulnerable groups and those without access to digital channels are not excluded. The effectiveness of the multistage process outlined in this paper will be determined by future user testing to confirm that it can deliver clear and actionable information to those in need.

The current rising momentum and expansion of digital technologies in the UHC era offers a significant opportunity to provide more equitable access to information globally. The *Your life, your health* resource is the first of its kind to be developed by WHO. It aims to consolidate technical and often disparate health information available to the general public in an integrated, understandable, and actionable way. The next step will be to focus on user testing of the resource prior to implementation at the global, regional, and country levels.

## Supplementary material

10.2196/57881 Multimedia Appendix 1 Global strategies, action plans, and guidance included in the evidence review.

10.2196/57881 Multimedia Appendix 2 Countries for which national health strategies were included in the evidence review.

10.2196/57881 Multimedia Appendix 3 “Actions and rights for healthy lives” consultation feedback form.

10.2196/57881 Multimedia Appendix 4 Digital cards adapted from the “Your life, your health: Tips and information for health and well-being” online resource.

## Abbreviations

DCP3: Disease Control Priorities 3rd Edition
mHealth: mobile health
UDL: Universal Design for Learning
UHC: universal health coverage
UN: United Nations
WHO: World Health Organization
