# Supplementary material for: “Your Life, Your Health: Tips and Information for Health and Well-Being”: Development of a World Health Organization Digital Resource to Support Universal Access to Trustworthy Health Information
Source: JMIR Form Res. 2025 Mar 6;9:e57881. doi: 10.2196/57881 (PMC11906094; doi:10.2196/57881)
Supplement: Multimedia Appendix 1 [file formative-v9-e57881-s001.docx]

**Supplementary Table 1.** Global strategies, action plans, and guidance included in the evidence review

| Global Strategy for Women’s, Children’s, and Adolescent’s Health  Global Strategy and Action Plan on Ageing and Health  Strategies Toward Ending Preventable Maternal Mortality (EPMM)  Every Newborn: An Action Plan to End Preventable Deaths  Framework for Nurturing Care for Early Childhood Development  Global Accelerated Action for the Health of Adolescents (AA-HA!)  Reproductive Health Strategy to Accelerate Progress towards the Attainment of International Development Goals and Targets  INSPIRE: Seven Strategies for Ending Violence Against Children  Global Vaccine Action Plan 2011-2020 | Accelerated Progress on HIV, Tuberculosis, Malaria, Hepatitis and Neglected Tropical Diseases  Global Action Plan for the Prevention and Control of Noncommunicable Diseases  Global Plan of Action to Strengthen the Role of the Health System to Address Interpersonal Violence  Global Strategy to Stop Health-Care Providers from Performing Female Genital Mutilation  Roadmap for Action: Integrating Equity, Gender, Human Rights and Social Determinants into the Work of WHO  Tackling NCDs: ‘Best Buys’ and Other Recommended Interventions for the Prevention and Control of NCDs  The Helsinki Statement on Health in All Policies. | Healthy Systems for Universal Health Coverage: A Joint Vision for Healthy Lives.  A Vision for Primary Health Care in the 21st Century: Towards Universal Health Coverage and the Sustainable Development Goals.  Draft WHO Global Strategy on Health, Environment and Climate Change  The Minsk Declaration: The Life-Course Approach in the Context of Health 2020  Global Strategy on People-Centered and Integrated Health Services  UNICEF Facts for Life  Timed and Targeted Counselling  The Lancet and BMJ series/collections on key interventions2 |
| --- | --- | --- |
