# Supplementary material for: “Your Life, Your Health: Tips and Information for Health and Well-Being”: Development of a World Health Organization Digital Resource to Support Universal Access to Trustworthy Health Information
Source: JMIR Form Res. 2025 Mar 6;9:e57881. doi: 10.2196/57881 (PMC11906094; doi:10.2196/57881)
Supplement: Multimedia Appendix 2 [file formative-v9-e57881-s002.docx]

**Supplementary Table 2.** Countries for which National Health Strategies were included in the evidence review


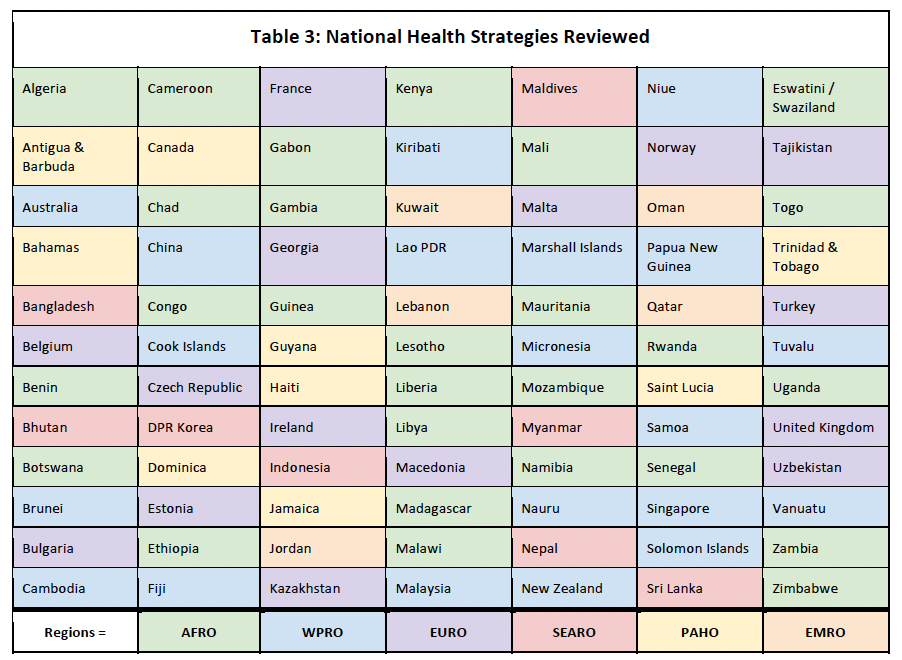


Analysis from Global Health Insights for *the Your Life, Your Health* Resource
