# Supplementary material for: “Your Life, Your Health: Tips and Information for Health and Well-Being”: Development of a World Health Organization Digital Resource to Support Universal Access to Trustworthy Health Information
Source: JMIR Form Res. 2025 Mar 6;9:e57881. doi: 10.2196/57881 (PMC11906094; doi:10.2196/57881)
Supplement: Multimedia Appendix 3 [file formative-v9-e57881-s003.docx]

Supplementary File 3.

| **‘Actions and Rights for Healthy Lives’**  **Consultation Feedback Form**  ‘Actions and Rights for Healthy Lives’ is a WHO public information resource. It draws on current health strategies, guidelines and evidence. It is people-centred and supports Primary Health Care, Universal Health Coverage and the Sustainable Development Goals. It uses health literacy principles to make information more accessible, understandable and actionable. It can help build awareness and dialogue for health. On its own, this resource is not a health communication or health promotion tool. Review and adaptation for different country contexts is important.  Please treat the draft resource as confidential and do not circulate it before it is officially published.  This feedback form has four sections:       Section 1 – Content and Priorities      Section 2 – Message Format and Clarity      Section 3 – Potential Uses, Potential Users      Section 4 – General Comments and Advice    While you are welcome to provide feedback and advice in all sections, you can skip entire sections if you do feel they match your area of interest or expertise. You are also able to skip individual questions throughout the form.  **About you**  Which country do you live in?   - Drop down list (UN list of countries) [https://www.un.org/en/member-states/index.html](https://protect-au.mimecast.com/s/F26YCwVLQmimw5N4hV8o-1?domain=un.org)     In which type of organization do you work?  Please select the option that best corresponds to the capacity in which you are responding.   - Government - UN or other multilateral organization - Non-governmental organization - Donor agency or foundation - Healthcare professional association - Private sector corporation - Small business/ self employed - Education, Academic/research/training institution - Personal - Other, please explain     At which geographical level do you work?   - National/ sub-national - Regional - Global/ HQ     In which WHO region are you located?   - African Region - Region of the Americas - South-East Asia Region - European Region - Eastern Mediterranean Region - Western Pacific Region   What is your area of expertise?   - Open ended   **Section 1 Content and priorities**  This section will be of greatest interest to those with specific content and/or life-stage expertise. It has been developed following an evidence synthesis of the leading preventable causes of mortality and morbidity by life-stage, and been reviewed by subject and/or life stage experts in WHO. The evidence synthesis may be viewed here.  I**f this is not your area of interest or expertise, you can move directly to section 2 by clicking here**  For each stage in the life course, there are a maximum of five evidence-based priority messages, together with information on key age-relevant health rights (questions 1.1 – 1.6). The information is provided in a tiered format, allowing the reader to obtain more information on each issue as desired. This age-specific content needs to be considered in conjunction with the generic sections on *finding and using health information* (question1.7) and *a healthy world* (question 1.8). This latter part of the resource recognises that many of the causes and consequences of illness and health are beyond the immediate control of the individual.  **In this section, we would particularly value your feedback on the choice of priority actions and rights, and the action statements. Please keep in mind that if you recommend inclusion of an alternative/additional priority, it would be most helpful if you could recommend which existing choice should be substituted.**  **If appropriate, you can move quickly from question to question by clicking on *Not my area of interest/expertise***  **1.1 Life stage: Family Planning, pregnancy and childbirth**  **Not my area of interest/expertise**  **Actions:** Using modern contraceptive methods; Planning for a healthy pregnancy and childbirth; Eating nutritious food; Keeping well; Connecting with health services.  **Rights:** Marriage; Having children; Non-discrimination; Making your voice heard  **1.2 Life stage: Newborn babies and children under-5 years**  **Not my area of interest/expertise**  **Actions:** Breastfeeding; Skin-to-skin contact; Sleeping safe; Keeping clean; Connecting to health services; Engaging and playing; Immunizing.  **Rights:** Legal identity; Children as individuals; Parents and carers; Government and community  **1.3 Life Stage: Older Children aged 5-9 years**  **Not my area of interest/expertise**  **Actions:** Helping children learn; Encouraging children to play and be active; Keeping children safe; Establishing healthy routines for life; Connecting to health services  **Rights:** Legal identity; Children as individuals; Parents and carers; Government and community  **1.4 Life Stage: Adolescence and Youth 10-24 years**  **Not my area of interest/expertise**  **Action:** Learning and developing skills; Having fun and healthy relationships; Staying safe; Keeping well; Connecting to health services  **Rights:** Marriage; Having children; Non-discrimination; Making your voice heard  **1.5 Life Stage: Early and middle adulthood 25-64 years**  **Not my area of interest/expertise**  **Actions:** Healthy eating; Healthy relationships and safe sex; Keeping well ; Sharing thoughts and feelings and responsibilities; Connecting to health services  **Rights:** Marriage; Having children; Non-discrimination; Making your voice heard  **1.6 Life Stage: Later adulthood and healthy ageing**  **Not my area of interest/expertise**  **Actions:** Healthy eating; Staying active; Staying social; Staying safe; Connecting to health services  **Rights:** Non-discrimination and protection from harm; Making your voice heard; Property  **1.7 Getting and Using Health information:**  **Not my area of interest/expertise**  **How to get health information:** Healthcare providers; Friends, family and the community; Media and internet  **How to use health information:** Using information to make decisions about your health and the health of your family; Using information to help others and the community.  **1.8 A healthy world:**  **Not my area of interest/expertise**  **People and sectors:** Individuals, families and communities; Health care providers; Education and training; Governments; Businesses and shops; Media; International organizations and partners  **SDGs and Rights**  **1.9 And finally, any other observations and advice on the content of the resource**  **Section 2: Messaging format and clarity**  **This section will be of greatest interest to those with health communication/health literacy interest and expertise, from both professional and consumer perspectives.**  **If this is not your area of interest or expertise, you can move directly to section 3 by clicking here**  **The main purpose of the resource is to offer vital health information directly to the public in a form that is accessible, understandable and actionable.**  **The information is provided in a tiered format, allowing the reader to obtain more information on each issue as desired. The content is age-specific and needs to be considered in conjunction with the sections on finding and using health information, and a healthy world. This latter section recognizes that many of the causes and consequences of illness and health are beyond the immediate control of the individual.**  **In this section, we would value your feedback on the communication structure and methods.**  **If appropriate, you can move quickly from question to question by clicking on *Not my area of interest/expertise***  **2.1 Structure of information presentation**:  We are seeking your advice and feedback on our use of tiered information. This approach has been adopted to provide a clear and simple initial message, and then to enable the person using the resource to find out more about the issue as they wish to.  **Not my area of interest/expertise**  **2.2 Clarity of messaging:**  We have tried to provide clearly written messages using plain language that is understandable and supports people in making healthier choices. We are seeking your advice and feedback on the tone, language, and volume of information in the different sections of the resource.  **Not my area of interest/expertise**  **2.3 Use of images:**  We have used a range of images to make the resource more personal and engaging for users. We are seeking your advice and feedback on the choice and style of images to support the document.  **Not my area of interest/expertise**  **2.4 Ease of use:**  We have developed this as an online resource to optimise accessibility. We are seeking your advice and feedback on the ease of use and overall functionality of the web site.  **Not my area of interest/expertise**  **2.5 Use of health literacy principles:**  We have developed the resource to provide accessible, understandable and actionable messages to a broad a cross section of people. We are seeking your advice and feedback on ways in which we can better support understanding, and enable practical action by those using the resource.  **Not my area of interest/expertise**  **2.6 And finally, any other observations and advice on the messaging and presentation of the resource**  **Section 3: Potential uses, potential users**  **This section will be of greatest interest to those who represent consumers, and those who can use the resource by working directly with individual health consumers and communities, as well as those supporting professional education and workforce development.**  **If this is not your area of interest or expertise, you can move directly to the Final comments section by clicking here**  **The main purpose of the resource is to offer vital health information directly to the public in a form that is accessible, understandable and actionable. The information is provided in a tiered format, allowing the reader to obtain more information on each issue as desired. The content is age-specific and needs to be considered in conjunction with the sections on finding and using health information, and a healthy world. This latter section recognizes that many of the causes and consequences of illness and health are beyond the immediate control of the individual.**  **In this section, we would value your feedback on potential uses and potential users of the resource.**  **If appropriate, you can move quickly from question to question by clicking on *Not my area of interest/expertise***  **3.1 Access:**  The resource has been developed to make vital health information accessible to as wide a variety of people as possible. We are seeking your advice and feedback on ways in which we can make the resource more accessible and more usable for a wide range of populations.  **Not my area of interest/expertise**  **3.2 Utility for frontline practitioners:**  We intend for the resource to be used directly by frontline health, education and other practitioners as a reliable source of information and education materials for their clients, students and communities. We are seeking your advice and feedback on ways in which we can make the resource as useful as possible for frontline practitioners.  **Not my area of interest/expertise**  **3.3 Adaptability**  We recognize that the utility of the resource will be influenced by the context in which it is to be used, and that for different communities it will need local adaptation and contextualization. We are seeking your advice and feedback on ways in which we can support local adaptation of the resource.  **Not my area of interest/expertise**  **3.4 Future Development:**  We see this as a living document that will need further development as evidence and priorities change. We are seeking your advice and feedback on ways in which we can continue to improve its relevance, accessibility and utility into the future.  **Not my area of interest/expertise**  **3.5 And finally, any other observations and advice on the utility of the resource**  **Section 4 General comments and advice**  Please provide any other observations and advice that you have not had the opportunity to make on the resource  Thank-you for participating in the 'Actions and Rights for Healthy Lives' consultation. If you would like to receive a summary of the results, please provide your email address below.  Email: ____________________________ |
| --- |
