# Supplementary material for: “Your Life, Your Health: Tips and Information for Health and Well-Being”: Development of a World Health Organization Digital Resource to Support Universal Access to Trustworthy Health Information
Source: JMIR Form Res. 2025 Mar 6;9:e57881. doi: 10.2196/57881 (PMC11906094; doi:10.2196/57881)

**Supplementary File 4.** Digital cards adapted from the ‘*Your life, your health: Tips and information for health and wellbeing’* online resource


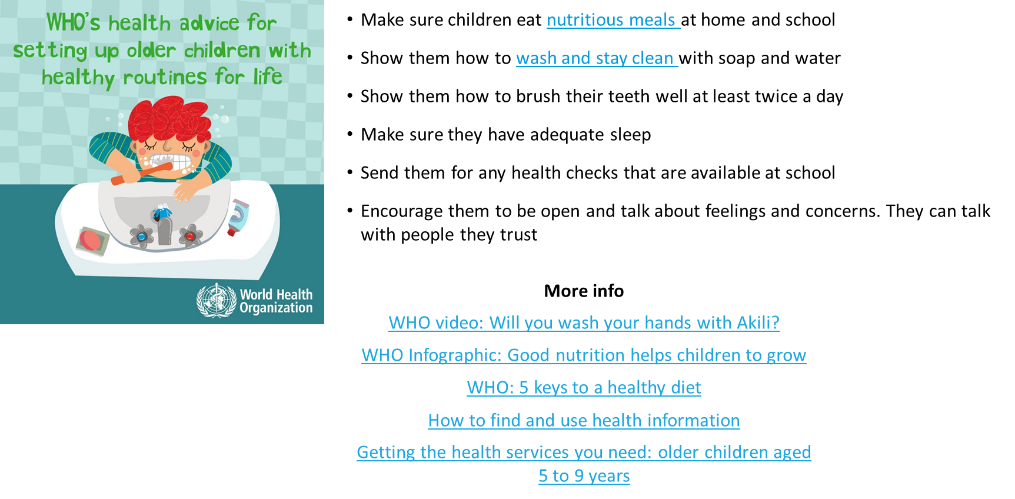

Supplement: Multimedia Appendix 4 [file formative-v9-e57881-s004.docx]
